# Supplementary material for: Volcanism and basalt weathering drove Ordovician climatic cooling
Source: Nat Commun. 2025 Dec 12;16:11475. doi: 10.1038/s41467-025-66316-4 (PMC12749037; doi:10.1038/s41467-025-66316-4)
Supplement: Supplementary file 1 — Supplementary Information [file 41467_2025_66316_MOESM1_ESM.pdf]

Supplementary Information

Volcanism and basalt weathering drove Ordovician climatic cooling

He Zhao[1], Lei Zhang[2]*, Thomas J. Algeo[2, 3, 4, 5], Zhengyi Lyu[2], Xiangdong Wang[2], Fang Hao[6]

[1] College of Marine Science and Technology, China University of Geosciences, Wuhan 430074, China
[2] State Key Laboratory of Geological Processes and Mineral Resources, China University of Geosciences, Wuhan 430074, China
[3] State Key Laboratory of Geomicrobiology and Environmental Changes, China University of Geosciences, Wuhan 430074, China
[4] Department of Geosciences, University of Cincinnati, Cincinnati, OH 45221-0013, USA
[5] State Key Laboratory of Oil and Gas Reservoir Geology and Exploitation, Chengdu University of Technology, Chengdu 610059, China
[6] National Key Laboratory of Deep Oil and Gas, China University of Petroleum (East China), Qingdao, Shandong, 266580, China

*Corresponding author: zhanglei_cug@sina.com (Lei Zhang)

This PDF file includes:

Supplementary Notes

S1. Intercalibrated biostratigraphic and chemostratigraphic frameworks;
S2. Preservation of conodont $\delta^{18}O$, derived SSTs, and their global comparisons;
S3. Preservation of Hg content and Hg isotopes in sedimentary rock;
S4. Baseline values and thresholds for determination of anomalous Hg enrichments in sedimentary rock;
S5. Comparisons between our present data and globally compiled Hg proxies and tuff layers;
S6. Regional seawater redox conditions;
S7. Paleotectonic and volcanic setting of study units, and potential global climatic response to regional volcanic arc systems;
S8. $^{87}Sr/^{86}Sr$ proxy for hydrothermal versus granite weathering fluxes;
S9. Early land plant evolution in the Ordovician Period;

Supplementary Figures 1 to 14;

Supplementary References

S1. Intercalibrated biostratigraphic and chemostratigraphic frameworks

The three study sections have well-established conodont and/or graptolite biostratigraphic frameworks. Previous studies established 21 conodont zones spanning the Nanjinguan to Baota formations (Fms) in the Huanghuachang section[1,2], identified 6 conodont zones from the Guniutan to Lingxiang Fms in the Chenjiahe section[3], and recognized 10 graptolite zones in the Wangjiawan section[4] (Supplementary Fig. 3). These biozones are correlative with the integrated Ordovician biozonation of South China[5].

Previous studies have documented a series of globally pronounced positive shifts in $\delta^{13}C_{carb}$ (i.e., Isotopic Carbon Excursion, ICE) throughout the Ordovician, such as the Middle Tremadocian ICE (i.e., TSICE), the Middle Darriwilian ICE (i.e., MDICE), the ICE during the Sandbian-Katian boundary transition (i.e., Guttenberg ICE, GICE), and Hirnantian ICE (i.e., HICE) [6]. Our newly generated $\delta^{13}C_{carb}$ data at Huanghuachang show no significant correlation to either $\delta^{18}O_{carb}$ ($r = +0.10$; $n = 256$; $p > 0.05$) or Mn/Sr ($r = +0.23$; $n = 50$; $p > 0.05$) (Supplementary Fig. 4), suggesting limited alteration of $\delta^{13}C_{carb}$ during diagenesis. $\delta^{13}C_{carb}$ data at Chenjiahe exhibit a moderate negative relationship to $^{18}O_{carb}$ ($r = -0.46$; $n = 38$; $p < 0.05$) but no correlation to Mn/Sr ($r = +0.38$; $n = 14$; $p > 0.05$), suggesting some alteration of $\delta^{13}C_{carb}$ during diagenesis. The newly generated $\delta^{13}C_{carb}$ curves for the Huanghuachang and Chenjiahe sections, as well as the published $\delta^{13}C_{carb}$ curve for the Wangjiawan section[7], show major excursions (e.g., TSICE, MDICE, and GICE) that are comparable with global representative $\delta^{13}C_{carb}$ curves (Supplementary Fig. 4). On the basis, we infer that the $\delta^{13}C_{carb}$ profiles of the Huanghuachang and Chenjiahe sections appear to preserve largely primary marine isotopic values.

Integrated biostratigraphic and carbon isotope chemostratigraphic data from the study sections, correlated with the Ordovician biostratigraphic framework in South China[5], collectively constrain the stage boundaries of Ordovician within these sections (Supplementary Fig. 5). In the Huanghuachang section, the base of the Tremadocian has been constrained near the bottom of the Nanjinguan Fm[5], where the *Cordylodus angulatus* Zone in the lower Nanjinguan Fm serves as a diagnostic fossil zone for the lower Tremadocian. The TSICE occurs within the Fenxiang Fm, indicating a middle to late Tremadocian age for this interval. The base of the Floian is constrained near the bottom of the Honghuayuan Fm[5]. The *Prioniodus honghuayuanensis* Zone within the Honghuayuan Fm indicates a middle Floian age. The base of the Dapingian (i.e., its GSSP) is located at 10.57 m above the base of the Dawan Fm, marked by the first appearance datum (FAD) of *Baltoniodus triangularis*[1]. The base of the Dapingian is also marked by the termination of the positive $\delta^{13}C_{carb}$ shift commencing in the lower Floian. The base of the Darriwilian is positioned near the top of the Dawan Fm[5]. The FAD of the *Lenodus variabilis* Zone in the lower Guniutan Fm indicates an early Darriwilian age. The base of the Sandbian is located within the *Pygodus anserinus* Zone in the Miaopo Fm. The base of the Katian occurs within the lower Baota Fm, approximately synchronous with the GICE. The base of the Hirnantian is roughly located within the Wufeng Fm, and the Ordovician-Silurian boundary is located near the bottom of the Longmaxi Fm[5].

In the Chenjiahe section, the *Yangtzeplacognathus protoramosus* Zone in the uppermost Guniutan Fm is diagnostic of the uppermost Darriwilian[3]. A positive $\delta^{13}C_{carb}$ shift in the Guniutan Fm forms the globally correlatable MDICE. The *Yangtzeplacognathus jianyensis* Zone within the Miaopo Fm indicates the Darriwilian-Sandbian boundary[5]. The FAD of *Hamarodus brevirameus* in the lower Baota Fm indicates an early Katian age.

In the Wangjiawan section, the base of the Hirnantian (i.e., its GSSP) is located within the upper Wufeng Fm, marked by the FAD of *Metabolograptus extraordinarius*[4]. The *Dicellograptus*

complexus Zone at the base of the Wufeng Fm indicates that the base of the Wangjiawan section is of late Katian age. The Ordovician-Silurian boundary (i.e., the top of the Hirnantian Stage) is located close to the base of the Longmaxi Fm, corresponding to the *Metabolograptus persculptus* Zone. The positive $\delta^{13}C_{carb}$ shift in the Wangjiawan section corresponds to the HICE.

S2. Preservation of conodont $\delta^{18}O$, derived SSTs, and their global comparisons

The color alteration indices (CAI) of the study specimens range from 1 to 3, indicating limited thermal alteration in the bioapatite structure and its oxygen isotopic composition[8-14]. Although conodonts are inhomogeneous in structure and chemical composition[8,15], conodont albid crown is the densest bioapatite tissue that are more preferences for extract (near-) primary seawater O isotope signals, thus was targeted for in-situ oxygen isotope analyses and reconstruction of Ordovician sea-surface temperatures (SSTs) in previous[8-14] and present studies. In addition, the in-situ O isotopic analysis was conducted using identical laser ablation spot diameter (20 μm) and ablation duration (80 s) to minimize systematic biases and further improve the reliability of trends in the reconstructed SST curve.

Another factor influencing the reliability of the SST reconstruction in this study is a major change in conodont taxa and their preferred habitats throughout the study interval. For conodont taxa, there is a shift in dominance from *Drepanodus* sp. in the Lower Ordovician to more diverse genera (including *Drepanoistodus* sp., *Scolopodus* sp., *Periodon* sp., *Tripodus* sp., and *Paroistodus* sp.) in the Middle Ordovician, followed by a return to more limited diversity dominated by *Ansella* sp. in the Upper Ordovician (Supplementary Fig. 6). *Drepanodus* sp. lived in the surface mixed layer of continental shelf watermasses[16] and was more widely distributed geographically than *Drepanoistodus* sp., which inhabited deeper subtidal areas[17]. *Scolopodus* generally lived nearby carbonate shoals of inner to outer platform facies[18], *Periodon* was most abundant in slope and outer platform deposits, and species such as *Tripodus laevis* were typical of shallow-marine inner-shelf facies[19]. *Paroistodus* sp. were pandemic but with a preference towards higher latitudinal areas[18]. *Ansella* sp. is a typical deep-water taxa[20]. Overall, there is a change toward deeper-water habitats correspond to decline in conodont-based SSTs, potentially accounting for the observed increase of ~4‰ in the $\delta^{18}O_{conodont}$ profile during the Early to Middle and Middle to Late Ordovician. However, given that our newly generated $\delta^{18}O_{conodont}$ profile matches global data well (Fig. 2), it is likely that ecological influences were limited and (near-)primary $\delta^{18}O_{conodont}$ signals were preserved in the study sections.

The change of paleolatitude in South China may affect the results of the reconstructed SSTs in the present study. Previous studies suggested that the location of South China gradually shifted from ~30°S during the Early Ordovician to the Equator during the Late Ordovician[21]. Therefore, the SST curve from South China may be affected by latitude and temperature gradients, which may cause deviations from the global average temperature, thus a much higher absolute in the reconstructed SSTs is expected (Supplementary Fig. 7).

The three major cooling episodes that we integrated are reinforced through comparing with previously published $\delta^{18}O_{conodont}$ data from other individual locations, using either gas isotope ratio mass spectrometry (GIRMS)[11,12], sensitive high resolution ion microprobe (SHRIMP)[10,14] or secondary ion mass spectrometry (SIMS)[9,13] (Fig. 2). Previous studies suggest a bias between SHRIMP and GIRMS conodont analyses, in which the former is systematically ~0.6–1.3‰ higher than the later[22], while none systematically bias between SHRIMP and SIMS analyses. To enhance consistency across different data sources during data compilation, we apply a minimum correction of +0.6‰ when converting GIRMS results to the SHRIMP standard.

The newly generated $\delta^{18}O_{conodont}$ curve exhibits positive excursions of ~2-3‰ during the LFCE and LDCE, as well as the Hirnantian Glaciation, which are consistent with global published $\delta^{18}O_{conodont}$ data[9,10,13] (Fig. 2). For example, the LFCE from the Laurentian margins and Argentine Precordillera[9,10], the LDCE from Laurentia and Tarim[9,13] and the Hirnantian cooling from the Laurentia and Gondwana[14]. An offset of ~1 to 1.5 ‰ between newly generated and previously published data following the LDCE may be attributed to multiple processes, including spatial difference in SSTs which may relate to paleolatitude, taxon-related effects in conodonts (as we discussed above), and/or biases during data compilation. For example, there is a consistent ~4–6 °C temperature excess (corresponding to $\delta^{18}O$ depletion of ~1–1.5‰) in South China relative to global averages during the late Early to Late Ordovician, and this discrepancy was minimal during the Floian-Sandbian but intensified during the Katian, likely reflecting stronger latitudinal thermal gradient effects as South China approached equatorial latitudes in the Late Ordovician.

Although the Hirnantian Glaciation interval was documented in the newly generated data, it was not clearly evident in the globally compiled LOWESS curve (Fig. 2A), probably due to the fact that the short-term, pronounced cooling had been obscured by data smoothing techniques. A previous proposed climate warming around the Katian-Hirnantian boundary[23,24] is not evident in our new records, possibly because of low data resolution in that interval of the compiled data, smoothing of the data masked the transient fluctuations, or the limited duration or geographic extent of the warming event. Two low $\delta^{18}O_{conodont}$ values at the top of the study sections indicate temporary warming conditions at the end of the Hirnantian Glaciation[24].

To explore the global significance of reconstructing SST curve and the documented three cooling episodes (i.e., LFCE, LDCE and Hirnantian Glaciation) in this study, we calculated SSTs following Eq. 4[8,25], and compared our SSTs to curves of the global oceanic average temperature[26] and oxygen-isotope derived temperature[27] (Supplementary Fig. 6). Based on paleotemperature calculation formula, our newly generated data and global compiled $\delta^{18}O_{conodont}$ data depict ~1.5 ‰, ~1.5 ‰ and ~2.5 ‰ increases in $\delta^{18}O_{conodont}$, thus drops of SST by ~7 °C, ~7 °C and ~10 °C during the LFCE, LDCE and Hirnantian Glaciation, respectively. The major drop in SST by ~7 °C during the LFCE is comparable in the timing and magnitude to temperature curve reported by Goldberg et al.[27]. The LFCE do not clearly shown in Scotese et al.[26] probably because the $\delta^{18}O_{conodont}$-based data mostly derived from low latitude area (~0 to 30°S) (e.g., Albanesi et al.[10], Liu et al.[28], and this study), whereas the later study intergraded temperatures data from both low and high latitudinal areas. For the LDCE, the revealed ~7 °C drop in SST is comparable to synchronously drop by ~4 °C in Goldberg et al.[27] and ~7 °C in Scotese et al.[26]. The Hirnantian Glaciation, even not clearly shown in global compiled $\delta^{18}O_{conodont}$ curve, was still revealed by four $\delta^{18}O_{conodont}$ peak values in the present study documenting a ~10 °C drop which correlates well to ~10 °C drop reported in Goldberg et al.[27] and ~5 °C drop in Scotese et al.[26].

Therefore, our newly acquired $\delta^{18}O_{conodont}$ data preserve (near-)primary changes in paleotemperature. The positive excursions of $\delta^{18}O_{conodont}$ record a protracted (25-Myr-long) climatic cooling trend that aligns with global data (Supplementary Fig. 6). This trend occurred in multiple stages, beginning around the Lower/Middle Ordovician boundary (~470 Ma), reinforced around the Middle/Late Ordovician boundary (~460 Ma), and culminating in the Hirnantian Glaciation (~445-444 Ma).

S3. Preservation of Hg content and Hg isotope signals in sedimentary rock

Regarding preservations of Hg in sedimentary rock, weathering can remove Hg signals in organic-rich sediment, for example, leading to loss of Hg up to ~90% in highly weathered shales, while degradation may alter the type and quality of organic matter, especially for samples with low hydrogen and high oxygen index values (e.g., Type II, equivalent to burial temperatures of ~60-180 °C), thereby affecting the Hg/TOC ratio[29]. Our study successions consist largely of carbonate rocks that underwent low degrees of weathering based on field observations, therefore, weathering is unlikely to have been a dominant influence on Hg/TOC ratios. In addition, the color alteration indices (CAI) of the extracted conodont specimens range from 1 to 3, equivalent to burial temperatures of ~60-200 °C[8], suggesting degradation of organic matter may have been an influence on Hg/TOC ratios. However, there is no clear difference in the CAI of conodonts between younger and older strata, indicating a relatively uniform level of thermal alteration throughout the study sections. In addition, TOC is strongly correlated with [Hg] in the Chenjiahe ($r = +0.77$; $n = 50$; $p < 0.001$) and Wangjiawan sections ($r = +0.49$; $n = 52$; $p < 0.001$) (Supplementary Fig. 11), suggesting degradation of organic matter and/or uncertainty of TOC analysis had a limited effect on Hg/TOC ratio in the two sections. At Huanghuachang, the correlation between TOC and [Hg] is weaker ($r = +0.35$; $n = 266$; $p < 0.001$), implying potential impacts on Hg/TOC ratios due to degradation of organic matter and/or uncertainty of TOC analysis in carbonate rocks (e.g., higher $CaCO_3$ content of carbonate rocks in the Tremadocian). Overall, positive excursions and peak values in the Hg/TOC profiles were not caused by low TOC content (< 0.2 wt.%)[30], suggesting validity of Hg/TOC ratio in tracing Hg enrichment intervals in the study sections.

The preservation of primary Hg isotope signatures in these sections is supported by multiple lines of evidence. Experimental constraints demonstrate that Hg-MDF and -MIF values remain stable below ~250 °C, with pressure enhancing Hg retention in sediments, and temperatures >250 °C may induce δ^{202}Hg enrichment (>0.1‰) through preferential light isotope loss, while MIF signatures persist even at 800 °C[31,32]. For the study samples, burial temperature < 200 °C was revealed from 1 to 3 in CAI values for conodont taxa from the study sections[33], suggesting limited thermal alterations of Hg isotopes. This thermal resilience validates the Hg-MDF and Hg-MIF signals in the study sections as primary seawater records. In addition, diagnostic consistency also emerges from: 1) Strong covariations between δ^{202}Hg and Δ^{199}Hg across the Middle-Upper Ordovician (Supplementary Fig. 10), patterns incompatible with temperature/pressure-driven remobilization[31,32]; 2) Stratigraphic coherence between Hg isotope cyclicity and independent proxies ($\delta^{18}O_{conodont}$ and $^{87}Sr/^{86}Sr_{conodont}$) from equivalent horizons (Fig. 2), whose synchronicity was attributed to depositional rather than diagenetic controls.

S4. Baseline values and thresholds for determination of anomalous Hg enrichments in sedimentary rock

To identify Hg anomalies in sedimentary rock and minimize false positives in Hg anomalies resulting from TOC and Hg analytical uncertainties, we have implemented rigorous reevaluation of anomaly signals by using multiple baseline approaches.

First, because carbonates exclude Hg during formation (relative to TOC in sediment), increasing $CaCO_3$ concentration can dilute the concentration of Hg in sediments, therefore, a carbonate-free Hg content ($Hg_{cf} = Hg / (1 - CaCO_3/100)$ is expected to better reflect actual Hg enrichment during deposition[34]. In the study sections, $CaCO_3$ content varies considerably between 0.1% and 99.4%, and there is a roughly increasing trend in Hg content and decreasing trend in

$CaCO_3$ content from Lower to Upper Ordovician (even there is no significant correlation between $CaCO_3$ and Hg), suggesting a potential carbonate dilution of Hg in study samples. Therefore, calculation of Hg_{cf} is applied to jointly evaluate Hg enrichment in study successions (Fig. 2E).

Second, following previous approach (e.g., Racki et al.[35]), we used median Hg content and Hg/TOC ratios of the study sections (Huanghuachang, Chenjiahe and Wangjiawan) as background values, which are 131 ppb and 76 ppm/%, respectively, for organic-rich clastic rocks (designated as baselines c, d), and 2.4 ppb and 70 ppm/%, respectively, for carbonate rocks (designated as baselines g, h) (Fig. 2D-F).

Third, given our study spans the whole Ordovician Period, we supplemented these with more broadly representative Phanerozoic-scale baselines derived from >3,500 samples[30], in which average Hg content are 62 ppb for shale and 34 ppb for carbonate rocks (baselines a, b), and 144 ppb/wt% for all sedimentary rock, and 72 ppb/wt% only for samples with TOC>0.2% (baselines e, f) (Fig. 2D-F).

Fourth, we further implemented Hg enrichment factor (Hg_{EF}) calculations following Racki et al.[35], defined as $Hg_{EF} = (Hg/TOC)_{sample}/(Hg/TOC)_{baseline}$. $Hg/TOC_{baseline}$ was set as 144 ppb/wt% for TOC ≤ 0.2%, and 72 ppb/wt% for TOC > 0.2% following[30]. The value of Hg_{EF} >3 is used as threshold to indicate substantial enrichment of Hg in sedimentary rocks (cf. Racki et al.[35]) (Supplementary Fig. 8).

S5. Comparisons between our present data and globally compiled Hg proxies and tuff layers

Globally compiled Hg content, paired Hg/TOC ratios and calculated Hg_{EF} values from publications (i.e., a total of 22 globally distributed sections/cores) all show plateau values above baselines/threshold during the middle Katian, the Hirnantian and the Ordovician-Silurian boundary (OSB) transition (e.g., in the Wangjiawan section), indicating reliable enriched Hg in sedimentary rock during these periods (Supplementary Fig. 8). Only Hg content mostly above the baselines, while Hg/TOC ratios and calculated Hg_{EF} mostly below the baselines/threshold, or the opposites (i.e., lower Hg content, and higher Hg/TOC and Hg_{EF} values), for example, during the Darriwilian, Sandbian (e.g., in the Nanyigou section, Tarim[36]), and early Katian, suggesting false Hg enrichment signals during these periods. Our present study shows more available Hg data for the Early to the early Late Ordovician, exhibiting plateau values of carbonate-free Hg content (Hg_{cf}, as documented in the main text), Hg/TOC ratios and Hg_{EF} all above baselines/threshold during the late Floian and the Sandbian, with the former interval is newly defined in the present study and the latter interval had not well been depicted in the previous studies (e.g., Liu et al.[36]) (Fig. 2 and Supplementary Fig. 8).

Similar to the Hg content, our newly generated Hg isotopes mostly focus on the Early to early Late Ordovician, thus filling in the major gaps for the global published data, which are mostly limited to the Middle-Late Ordovician (especially the OSB transition). Studies on integration of $\Delta^{199}Hg$ data reveals that LOWESS-smoothed $\Delta^{199}Hg$ trend based on our study sections alone closely mirrors the global composite trend based on both new and published data (Fig. 2 and Supplementary Fig. 8), both displaying long-term decreasing trend (from ~+3 to ~–2‰) during the Early to early Middle Ordovician, followed by an increasing trend to ~0 to –0.1‰ in the Late Ordovician to the OSB transition.

LOWESS-smoothed $\delta^{202}Hg$ curves between the study sections and a compilation with previous published data also shows comparable trends, marked by negative values (from ~–3 to –1‰) with minor variation during the Early Ordovician, followed by a major rise to positive values (from ~0 to +1‰) during the Middle to early Late Ordovician before decreasing to negative values

(from ~–2 to 0‰) during the mid-Late Ordovician to OSB transition. Therefore, the identified Δ^{199}Hg and δ^{202}Hg signals through the Ordovician in the study sections represent a global pattern.

We additionally compiled previously published volcanic tuff (or called bentonite/volcanic ash) data from these 22 globally distributed sections/cores, predominantly from the Middle and Upper Ordovician, where paired Hg measurements had been figured out (Supplementary Fig. 8). We noticed that Huff et al.[37] once reported volcanic tuff records across the Lower to Upper Ordovician in multiple continentals, however, the corresponded Hg enrichment is unclear. To enhance global representativeness, we compared our compiled data to the volcanic tuff distribution dataset[37] (Supplementary Figure 13). The compilation of these published data shows the most active period of volcanic activity and tuff deposition during the Late Ordovician, moderate during the Middle Ordovician, while minimal during the Early Ordovician. The volcanic tuff layers preserved in the study sections, i.e., one layer in the upper Dawan Fm (of the Middle Ordovician age), and one layer in the Miaopo Fm and five layers in the upper Wufeng Fm (of the Late Ordovician age) (Supplementary Fig. 2), is overall comparable to the regional[38,39] and global patterns[37,40] (Supplementary Figure 13). Moreover, the temporal distribution of the volcanic tuff layer corresponds well with globally prominent Hg anomalies in the Late Ordovician, which further support volcanic origin Hg enrichment in sedimentary rock during the Ordovician.

S6. Regional seawater redox conditions

We explore changes in regional seawater redox conditions using Ce anomaly (Ce/Ce*), C_{org}/P ratios, U_{EF} and Mo_{EF}. Under oxic water conditions, the Ce^{4+} cation separates from other REEs (3+ cations), causing Ce enrichment and higher Ce/Ce* in sediments, whereas depleted Ce content and lower Ce/Ce* in seawater and carbonate minerals (e.g., carbonate fraction of bulk sample)[41]. C_{org}/P ratios are particularly valuable for evaluating redox conditions in carbonate facies, where the scarcity of organic matter generally restricts absorption of trace metals, with values of <50, ~50–100, and >100 indicative of oxic, suboxic, and anoxic environments, respectively[42]. Under reducing conditions, U and Mo remain insoluble and are preferentially enriched in the sediment[43]. In comparison, the enrichment of U begins at the Fe(III)/Fe(II) redox threshold, which corresponds to suboxic conditions. Meanwhile, Mo becomes reactive and binds to particles in the presence of aqueous hydrogen sulfide, indicating euxinic conditions. As a result, under suboxic to anoxic conditions, we observe lower U_{EF} and Mo_{EF} values, approximately 3–10 and 5–50 respectively, while under euxinic conditions, these values are significantly higher, exceeding 10 and 50 respectively.

In the present study, Ce/Ce^*_{carb} shows an overall decreasing trend from ~1 in the Nanjinguan to Fenxiang Fms, to ~0.8-1 in the Honghuanyuan to Guniutan formations, before drop to ~0.6-0.8 in the Miaopo to Linxiang Fms (Supplementary Fig. 9), indicating suboxic-anoxic conditions during the Tremadocian, and suboxic to oxic seawater during the Floian to Katian Stages. The overall pattern of oceanic oxygenation during the Early to Late Ordovician is further supported by a decreasing trend in C_{org}/P profile from ~50 to <10 mol/mol in the Nanjinguan to Honghuayuan Fms, and overall low values <10 mol/mol in the Dawan to Linxiang Fms. The lower U_{EF} and Mo_{EF} values (mostly < 3) in the Miaopo Fm confirmed oxic seawater conditions during the Middle-Late Ordovician boundary transition. Both U_{EF} and Mo_{EF} values show larger fluctuations, ~5–60 and ~5–120, respectively, at the Ordovician-Silurian transition, suggesting a more reducing seawater to anoxic-sulfidic conditions during the LOME.

S7. Paleotectonic and volcanic setting of study units, and potential global climatic response to regional volcanic arc systems

The study units were situated within the platform-slope-basin depositional system located between the Yangtze Platform of South China and the adjacent Cathaysia Block during the Ordovician. Along the margin of the Cathaysia Block proximal to the Jiangnan Slope, a series of NE-SW-trending (in present-day orientation here and below) arc belts developed. This platform-slope-basin system extensively records the deposition of potassium-rich bentonites (K-bentonites, that is, volcanic tuff) especially during the Late Ordovician Hirnantian stage, while reports of K-bentonites from other Ordovician epochs are relatively scarce (Su et al.[44]; this study). This disparity may be related to differences in geological preservation and research intensity. Paleogeographic reconstruction based on bed thickness measurements of the K-bentonites from multiple sections across the Yangtze region indicates that Hirnantian K-bentonite deposition was nearly ubiquitous throughout the Yangtze region. The affected area is roughly estimated to have covered ~0.5 million km^2[44]. Geochemical analyses reveal that these K-bentonites originated from volcanic eruptions in a collisional or accretionary zone tectonic setting[45,46]. The composition of the parent magma ranges from trachyandesite to rhyodacite, with some rhyolite, further indicating an origin in volcanic-arc and syn-collision to within-plate tectonomagmatic settings along an active continental margin. Their distribution correlates with cratonward-migrating black shales and flysch during a tectophase cycle, directly linking K-bentonite formation to northwestward arc-continent collision and accretion between the Cathaysia Block and Yangtze Craton during the Ordovician-Silurian boundary transition[44,47]. Our study units are located ~600–800 km from the volcanic vents along the margin of the Cathaysia Block, placing them at the periphery of the K-bentonite depositional extent.

Regional volcanic arc systems can generate global climatic effects. Global chemical weathering of continental volcanic arcs plays as rapid drawdown of CO_2 tied to arc weathering stabilizes surface temperatures over geological time[48]. Besides, probably act as one of the most important trajectories, reworking of accreted carbonate platforms located in mature continental arcs (i.e., crustal carbonate) is an important source of volcanic carbon during supercontinent formation and breakup[49,50]. Lee et al.[51] proposed climatic cooling effect of island arc (e.g., Neogene) and warming effect of continental arc (e.g., Cretaceous-Paleogene), which were associated with carbon fixation/release sequestered by global crustal carbonate reservoirs during assembly/dispersal of continents, therefore, long-term (>50 Myr) greenhouse-icehouse oscillations may be linked to fluctuations between continental- and island arc–dominated states. McKenzie et al.[52] further demonstrated continental volcanic arc emissions of CO_2 as the principal driver of long-term (multimillion-year time scales) icehouse-greenhouse variability over the past ~720 Myr, in which widespread continental arcs correspond with prominent early Paleozoic and Mesozoic greenhouse climates, whereas reduced continental arc activity corresponds with icehouse climates of the Cryogenian, Late Ordovician, late Paleozoic, and Cenozoic. Other evidence supports the mechanism, for example, extinction of Neo-Tethyan volcanic arcs (i.e., continental volcanic arc) is largely synchronous with phases of CO_2 reduction during climate cooling throughout the early to middle Cenozoic; enhanced volcanism and CO_2 emissions due to unloading of active magmatic provinces on continents during the last deglaciation[53].

S8. $^{87}Sr/^{86}Sr$ proxy for hydrothermal versus granite weathering fluxes

The $^{87}Sr/^{86}Sr_{conodont}$ profile of the present study is consistent with coeval data from Laurentia (United States)[54] and a global database[55], marked by an initial value of ~0.709 with a slightly decreasing trend during the earliest Tremadocian to middle Darriwilian, followed by an accelerated decline during the late Darriwilian to late Sandbian, stabilizing at ~0.708 in the Katian.

Continental weathering and marine hydrothermal activity are the dominant influences on seawater $^{87}Sr/^{86}Sr$ composition. The $^{87}Sr/^{86}Sr$ proxy is commonly used to infer the relative inputs of mantle versus continental crustal strontium[54]. The marine hydrothermal flux lowers seawater $^{87}Sr/^{86}Sr$, whereas the continental weathering flux increases it, depending on the type of rock being weathered. If granitic sources are dominant, the input flux from continental weathering will elevate seawater $^{87}Sr/^{86}Sr$ values, whereas a larger flux from basaltic rocks will decrease them. Furthermore, if there is no major change in the composition of the continental rocks, intensified weathering generally corresponds to rising seawater $^{87}Sr/^{86}Sr$ values.

The shift in the type of the exposed terrestrial rocks could have been a determining factor for the decline of $^{87}Sr/^{86}Sr$ during the Ordovician. The secular trends of decreasing $^{87}Sr/^{86}Sr$ suggest slightly stronger hydrothermal versus granite weathering fluxes into the ocean during the Tremadocian to middle Darriwilian, intensification hydrothermal versus granite weathering fluxes during the late Darriwilian to late Sandbian, and a steady condition during the late Sandbian to late Katian[12]. Hg geochemistry in this study indicates that volcanic activity was relatively subdued in the Early Ordovician but began to escalate during the Early-Middle Ordovician, which may reflect minor exposure of basalt during the Early Ordovician, followed by a pronounced increase at the transition to the Middle Ordovician, offering a coherent explanation for the two distinct declines in amplitude of marine $^{87}Sr/^{86}Sr$ records.

S9. Early land plant evolution in the Ordovician Period

Spatiotemporal changes of the earliest plants during the Ordovician were reviewed by Algeo et al.[56]: Earliest cryptospores from the Zanjon Fm of Argentina, dating to the Dapingian (early Middle Ordovician) at ~470-467 Ma[57-59]; Slightly younger cryptospore finds from the Czech Republic (mid-Darriwilian; ~464 Ma)[60,61] and Saudi Arabia (late Darriwilian; ~460 Ma)[62,63]; Other younger cryptospore comes from Sweden (Katian) in the Baltica[64]; The oldest known glomalean fungus is from Wisconsin, dating to the late Sandbian (~454 Ma)[65]; The oldest known mesofossils of sporangia are from the Ghaba-1 borehole in Oman and Saudi Arabia, dating to the mid-Katian (~449 Ma)[66,67]; The oldest known laevigate trilete spores are from Turkey, dating to the Hirnantian (~445 Ma)[67,68]. Although the fossil record of early bryophytes is highly incomplete, these finds suggest that bryophytes appeared around the beginning of the Middle Ordovician and subsequently spread and diversified during the Middle to Late Ordovician, establishing a community of photosynthesizers and decomposers by the end of the Ordovician.

Supplementary Figures

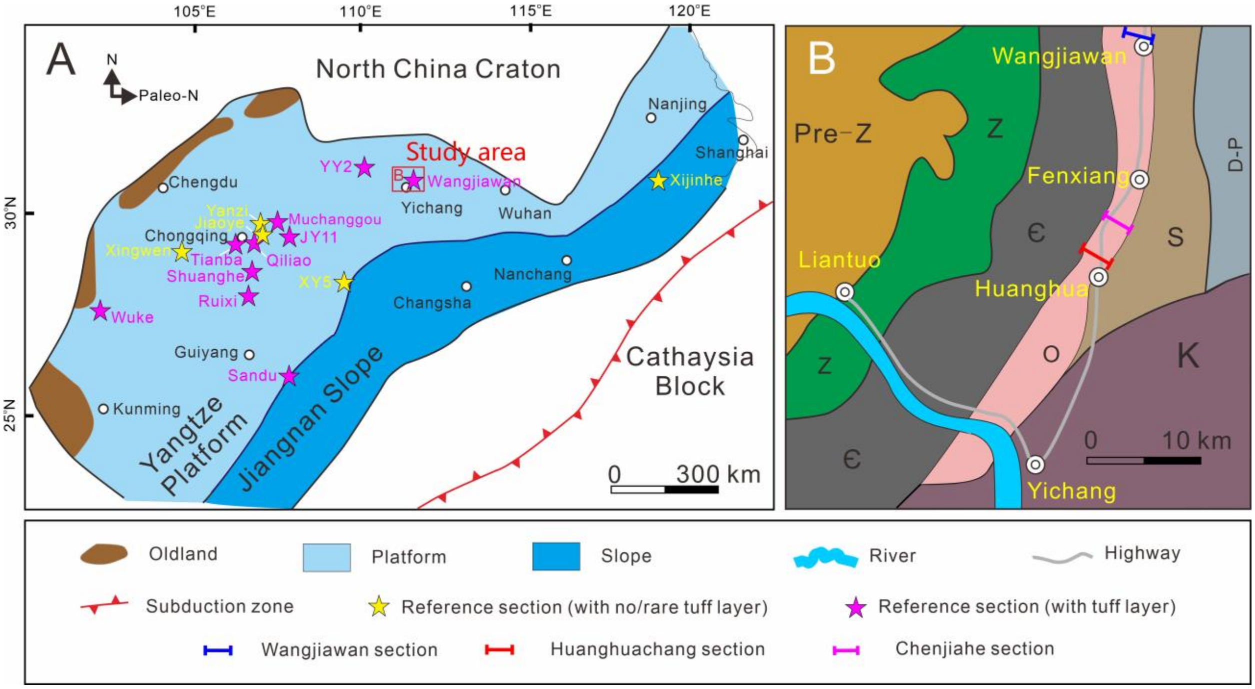

Supplementary Figure 1. Location of study sections and reference sections in South China. (A) Early to Middle Ordovician paleogeography of the South China Craton; (B) Geological map of Yichang area, showing the locations of the Huanghuachang, Chenjiahe and Wangjiawan sections. Panels A and B are modified from Zhang et al.[33]. In panel A, the subduction zones between the Cathaysia and Yangtze blocks are modified from Su et al.[44]. Z = Sinian, Є = Cambrian, O = Ordovician, S = Silurian, D = Devonian, P = Permian, K = Cretaceous.

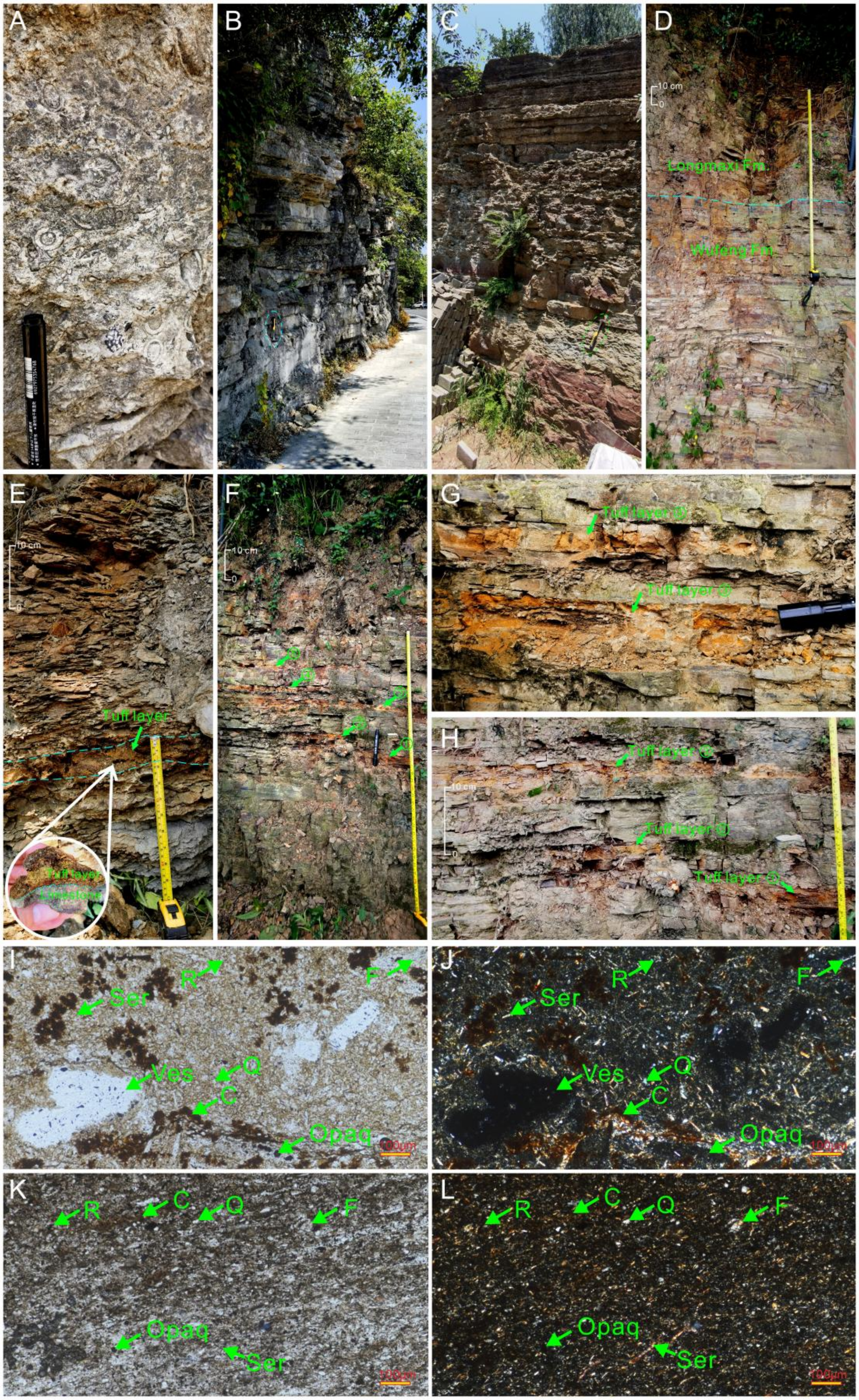
A
B
C
D
10 cm
0
Longmaxi Fm.
Wufeng Fm.
E
10 cm
0
Tuff layer
F
10 cm
0
G
H
10 cm
0
I
R
F
Ser
Ves
Q
C
Opaq
J
R
F
Ser
Ves
Q
C
Opaq
K
R
C
Q
F
Opaq
Ser
L
R
C
Q
F
Opaq
Ser

Supplementary Figure 2. Field photographs of typical lithologies (including tuff layers) in the study sections, with corresponding thin-section photomicrographs of representative volcanic tuff layers. (A) Reef limestone in the upper part of the Honghuayuan Formation, Huanghuachang section. (B) Alternating thin-bedded limestone and mudstone of the Dawan Formation, Huanghuachang section. (C) Purple-red marl of the Guniutan Formation in Chenjiahe section. (D) Clastic rocks near the boundary of the Wufeng-Longmaxi Formation in Wangjiawan section. (E) A tuff layer (~4 cm thick) within calcareous mudstone in the middle part of the Dawan Formation, Huanghuachang section. (F-H) Five tuff layers (numbered 1 to 5 from bottom to top, with thicknesses of ca. 2 cm, 2 cm, 4 cm, 2.5 cm, and 1 cm, respectively) within the Wufeng Formation, Wangjiawan section. (I-J) Thin-section photomicrographs of volcanic tuff layers in Dawan Formation and (K-L) 3th tuff layer in Wufeng Formation. Panel I shows the plane-polarized light image, while Panel J displays the corresponding cross-polarized light image of the same field of view. Panels K and L are related in the same manner. Abbreviations: Q = Quartz; R = Rock fragment; F = Feldspar; C = Clay mineral; Ser = Sericite; Opaq = Opaque mineral; Ves = Vesicle.

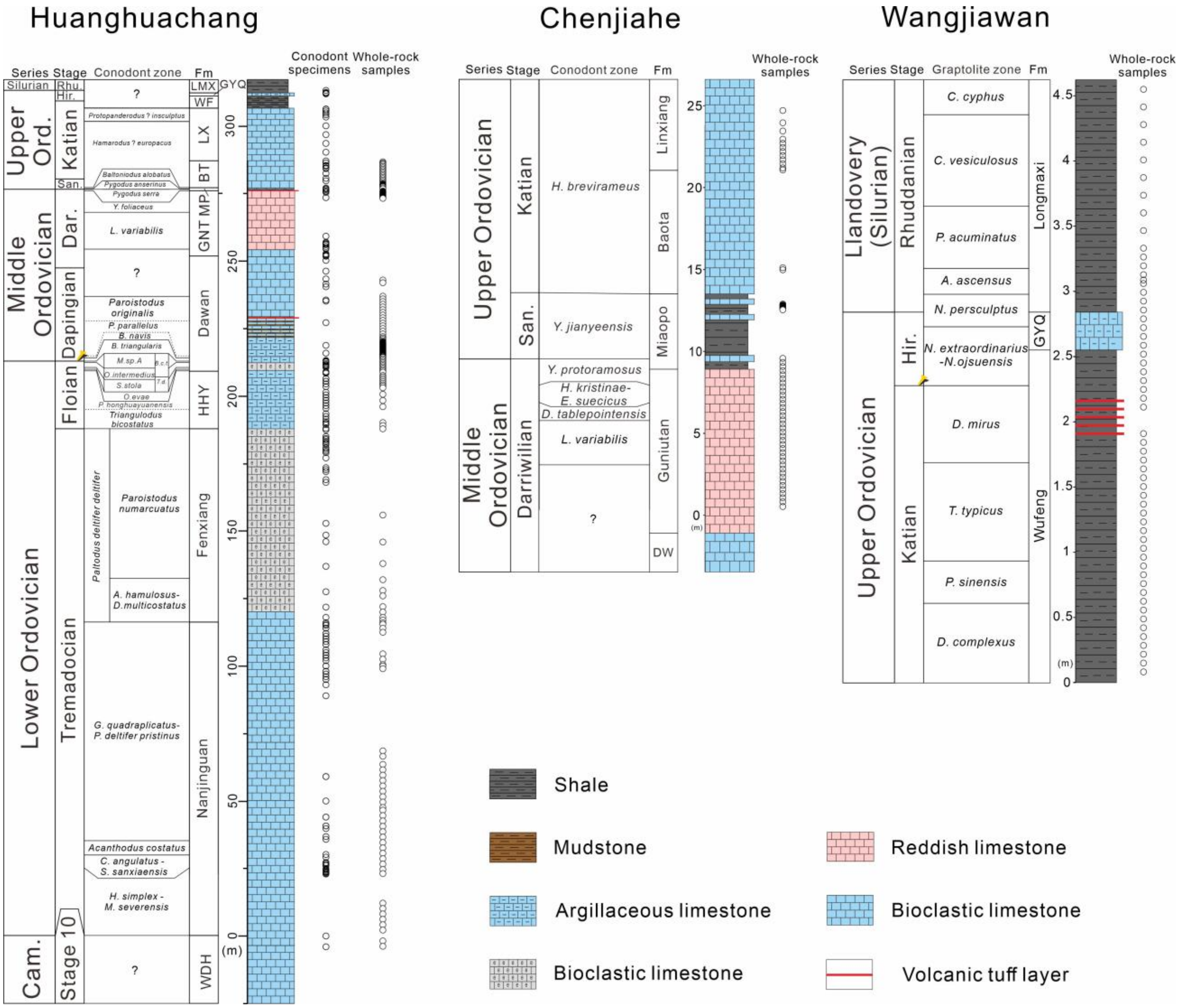

Supplementary Figure 3. Biozonation and lithostratigraphy of the Huanghuachang, Chenjiahe and Wangjiawan sections. Conodont zonations in the Huanghuachang and Chenjiahe sections are from Wang et al.[1-3]. Graptolite zonation of the Wangjiawan section is from Chen et al.[69]. Cam. = Cambrian; Dar. = Darriwilian; Hir. = Hirnantian; Ord. = Ordovician; San. = Sandbian; Fm = Formation; WDH = Wuduhe; HHY = Honghuayuan; GNT = Guniutan; MP = Miaopo; BT = Baota; LX = Lingxiang; WF = Wufeng; GYQ = Guanyinqiao; LMX = Longmaxi. The "Golden Spikes" show the locations of GSSPs in the Huanghuachang and Wangjiawan sections.

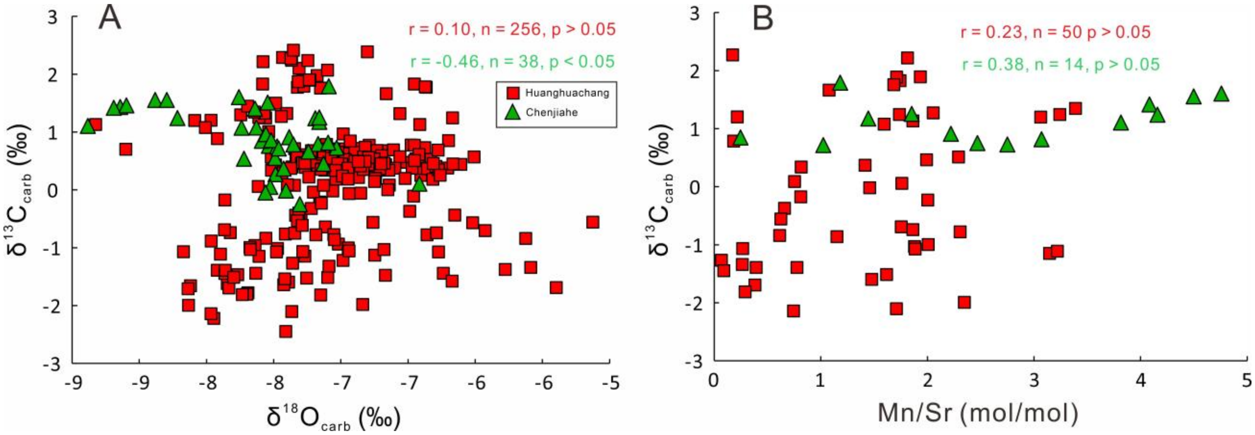

Supplementary Figure 4. Crossplots of $\delta^{13}C_{carb}$ vs. $\delta^{18}O_{carb}$ (A) and Mn/Sr (B) for the study sections.

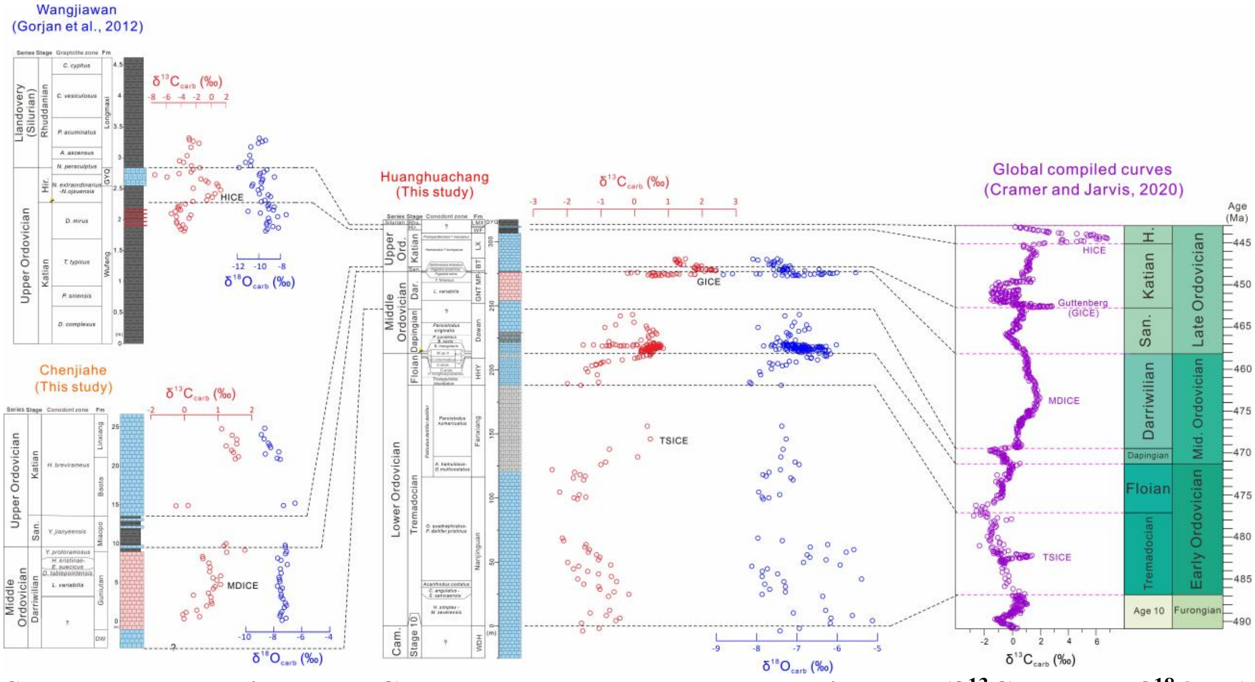

Supplementary Figure 5. Carbonate carbon and oxygen isotope ($\delta^{13}C_{carb}$ and $\delta^{18}O_{carb}$) profiles of the study sections, and comparisons to published global $\delta^{13}C_{carb}$ data. $\delta^{13}C_{carb}$ and $\delta^{18}O_{carb}$ data for Wangjiawan are from Gorjan et al.[7]. The globally compiled $\delta^{13}C_{carb}$ data are from Cramer and Jarvis[6]. TSICE = Middle Tremadocian positive $\delta^{13}C$ excursion; MDICE = Middle Darriwilian positive $\delta^{13}C$ excursion; GICE = Guttenberg positive $\delta^{13}C$ excursion; HICE = Hirnantian positive $\delta^{13}C$ excursion. For other abbreviations refer to Supplementary Fig. 3.

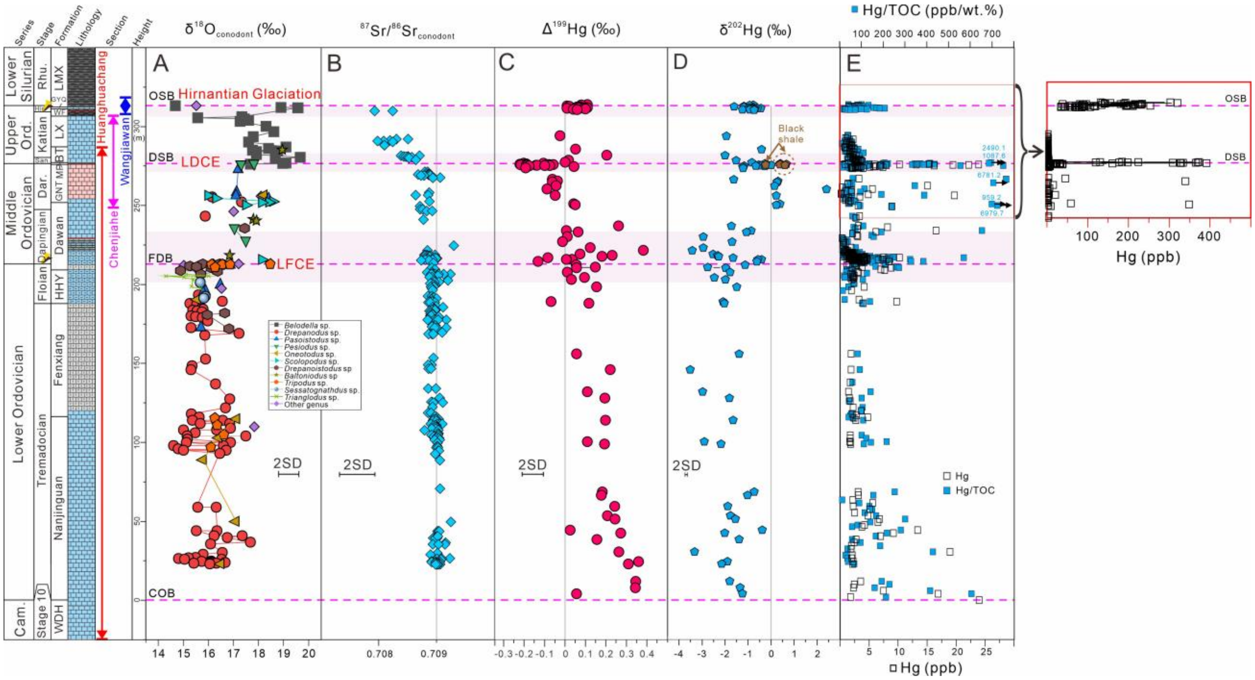

Supplementary Figure 6. Geochemical profiles of the study sections. (A) In-situ conodont oxygen isotopes ($\delta^{18}O_{conodont}$). (B) In-situ conodont strontium isotopes ($^{87}Sr/^{86}Sr_{conodont}$). (C-D) Mercury isotopes ($\Delta^{199}Hg$ and $\delta^{202}Hg$). (E) Hg content and its ratio to total organic carbon (Hg/TOC). COB = Cambrian-Ordovician boundary; FDB = Floian-Dapingian boundary; DSB = Darriwilian-Sandbian boundary; OSB = Ordovician-Silurian boundary. About a third of Hg/TOC data for the upper Honghuayuan and Longmaxi formations, as well as all $\Delta^{199}Hg$ and $\delta^{202}Hg$ data for the Wangjiawan section, are from Gong et al.[70]. Light pink fields represent cooling episodes as revealed from Figure 2: LFCE = Late Floian cooling event, LDCE = Late Darriwilian cooling event. For other abbreviations refer to Supplementary Fig. 3.

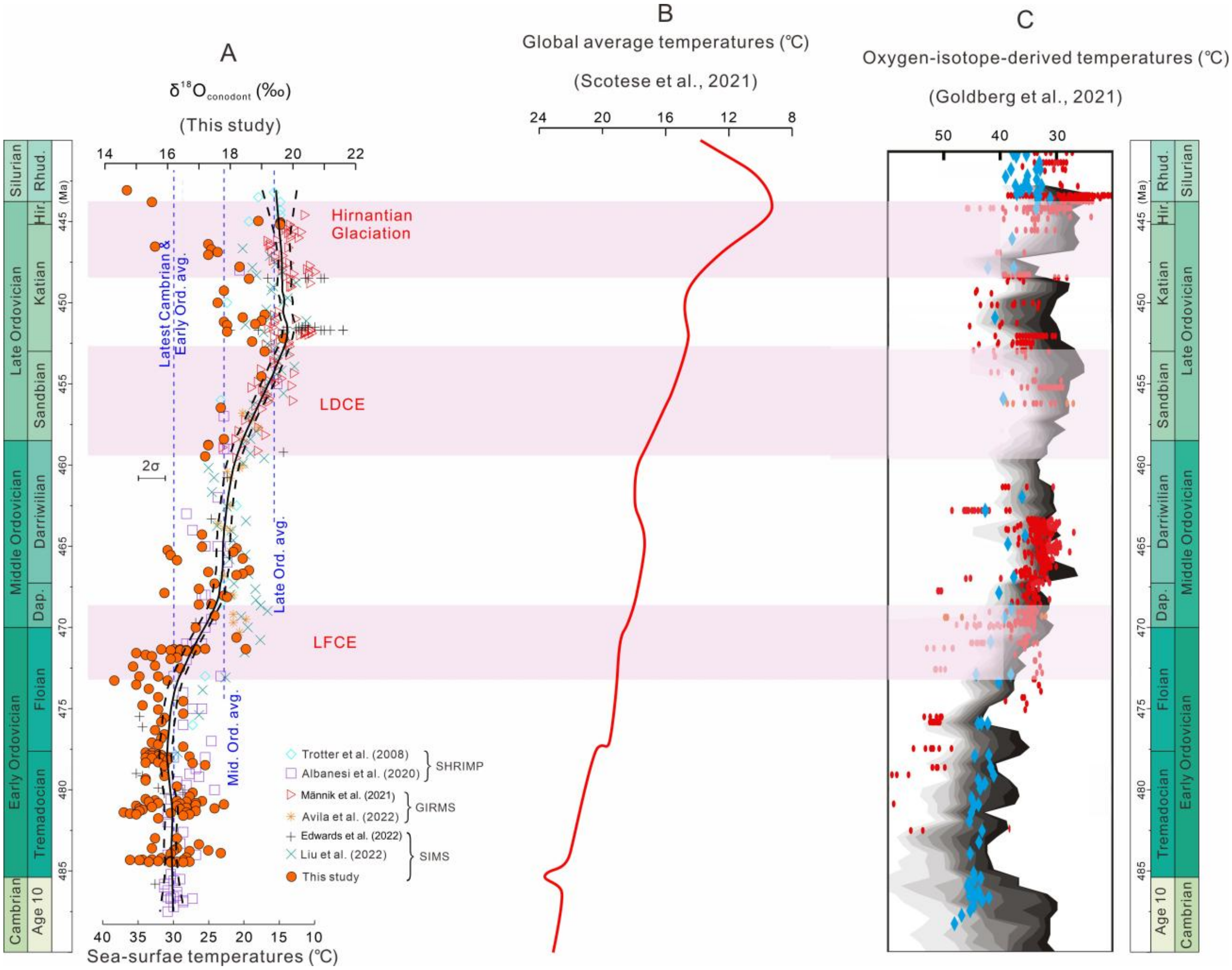

Supplementary Figure 7. Comparisons of Ordovician paleotemperature curves. (A) Conodont oxygen isotopes ($\delta^{18}O_{conodont}$) and calculated sea-surface temperatures based on Eq. 4 from Zhang et al. and Pucéat et al.[8,25]. (B) Global average temperature is from Scotese et al.[26], which was obtained by summing the latitudinal temperatures along the corresponding pole-to-equator temperature gradient curve. (C) Oxygen-isotope-derived temperatures are from Goldberg et al.[27]. Panel A is identical to Figure 2A. In panel C, the shading denotes the deciles of all global bulk carbonate oxygen isotope data to indicate the spread of the data. The darker regions in panel C correspond to the more ^{18}O-enriched (lower temperature) portion of the distribution, which corresponds to lesser degrees of diagenetic alteration. Small red points are temperature from brachiopod $\delta^{18}O$, and blue diamonds are temperature from conodont apatite $\delta^{18}O$ in Goldberg et al.[27].

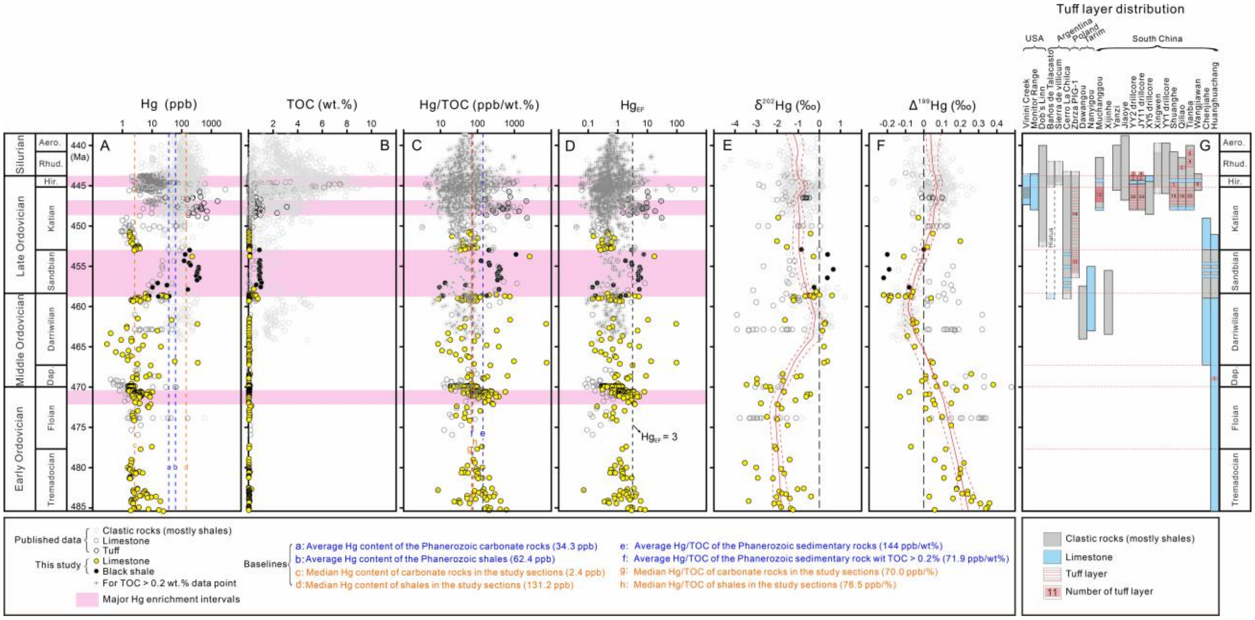

Supplementary Figure 8. Global Hg geochemical compositions of marine sediments and tuff layer distributions from the earliest Ordovician to early Silurian. (A) Hg content, (B) Total organic carbon (TOC) content, (C) Ratio of Hg content to total organic carbon (Hg/TOC), (D) Enrichment factor of Hg (Hg_{EF}), (E) δ^{202}Hg, (F) Δ^{199}Hg, (G) Spatiotemporal distributions of tuff layer. In panels A and C, mean/median Hg content and Hg/TOC ratio of Phanerozoic sedimentary rock were adopted as baseline values (dashed blue lines) for joint evaluation of Hg enrichment episodes, including average Hg content of Phanerozoic carbonate rocks (line a, 34 ppb) and shale (line b, 62 ppb)[30], median Hg content of our studied carbonate rocks (line c, 2.4 ppb) and organic-rich clastic rocks (line d, 131 ppb), and average Hg/TOC ratio of Phanerozoic sedimentary rock (line e, 144 ppb/wt%) and sedimentary rock of TOC > 0.2% (line f, 72 ppb/wt%)[30], and median Hg/TOC ratios of our studied carbonate rocks (line g, 70 ppb/%) and organic-rich clastic rocks (line h, 76 ppb/%). In panel D, Hg enrichment factor (Hg_{EF}) is calculated following the equation $Hg_{EF} = (Hg/TOC)_{sample}/(Hg/TOC)_{background}$)[35], where $(Hg/TOC)_{background}$ is adopted as 144 ppb/wt.% for the average Phanerozoic sedimentary rock and 71.9 ppb/wt.% for sedimentary rock of TOC > 0.2%[30]. $Hg_{EF} > 3$, along with Hg/TOC and Hg contents higher than baseline values, is regarded as a positive Hg anomaly. In panels E-F, the dashed and solid red curves are LOWESS fits, based on a smoothing factor of 0.2, incorporating both newly generated and published data. In panel G, vertical stripes indicate uncertain age ranges due to lacking biostratigraphic constrain. The published Hg geochemical data and total organic carbon (TOC) are archived in Figshare.

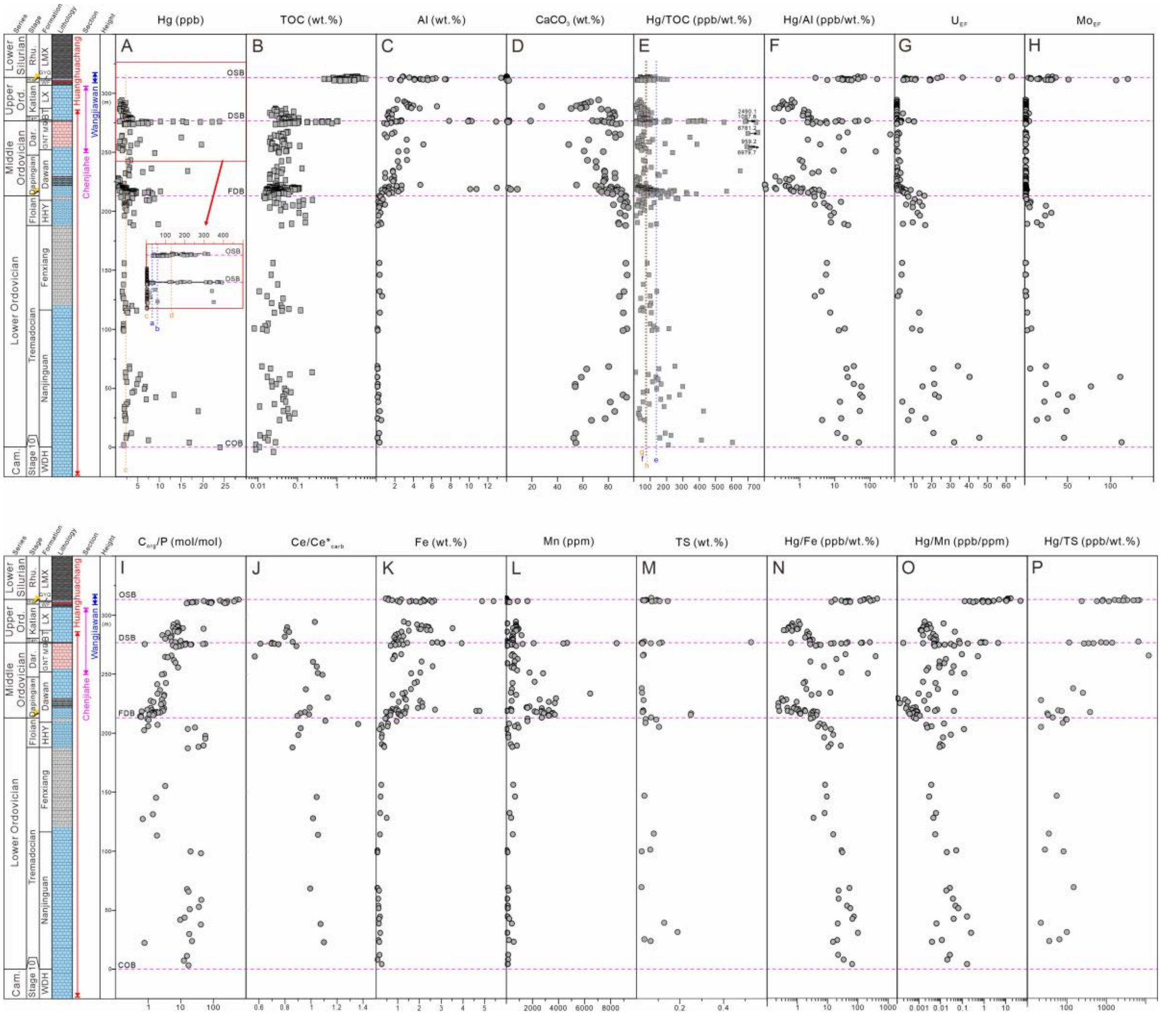

Supplementary Figure 9. Geochemical profiles of the study sections. (A) Mercury content (Hg, ppb); (B) total organic carbon content (TOC, wt.%); (C) aluminum content (Al, wt.%); (D) Carbonate content ($CaCO_3$, wt.%); (E) ratio of mercury to total organic carbon content (Hg/TOC, ppb/wt.%); (F) ratio of mercury to aluminum content (Hg/Al, ppb/ wt.%); enrichment factors of (G) uranium (U_{EF}) and (H) molybdenum (Mo_{EF}); (I) ratio of organic carbon content to phosphorus content (C_{org}/P, mol/mol); (J) Cerium anomaly of carbonate fraction (Ce/Ce^*_{carb}); (K) iron content (Fe, wt.%); (L) manganese content (Mn, wt.%); (M) total sulfur content (TS, wt.%); (N) ratio of mercury to iron content (Hg/Fe, ppb/wt.%), (O) manganese content (Hg/Mn, ppb/wt.%), and (P) total sulfur content (TS, wt.%). Note that only Ce/Ce^*_{carb} are specifically for carbonate fraction, and others are for bulk carbonate rock. COB = Cambrian-Ordovician boundary; FDB = Floian-Dapingian boundary; DSB = Darriwilian-Sandbian boundary; OSB = Ordovician-Silurian boundary. In panels A and E, average Hg content of Phanerozoic carbonate rocks (line a, 34 ppb) and shale (line b, 62 ppb)[30], median Hg content of our studied carbonate rocks (line c, 2.4 ppb) and organic-rich clastic rocks (line d, 131 ppb), and average Hg/TOC ratio of Phanerozoic sedimentary rock (line e, 144 ppb/wt%) and sedimentary rock of TOC > 0.2% (line f, 72 ppb/wt%)[30], and median Hg/TOC ratios of our studied carbonate rocks (line g, 70 ppb/%) and organic-rich clastic rocks (line h, 76 ppb/%) are adopted as baseline values (dashed blue lines) for jointly evaluation of Hg enrichment intervals.

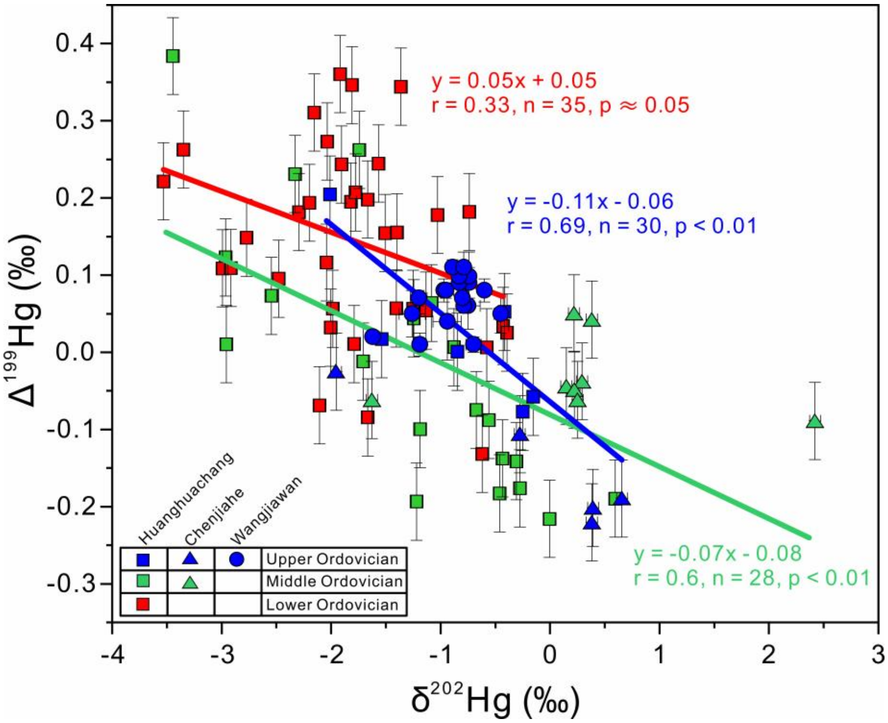

Supplementary Figure 10. Crossplots of Δ^{199}Hg vs. δ^{202}Hg for the study sections. The vertical bars represent standard deviations (2SD) for Δ^{199}Hg, and 2SD for δ^{202}Hg is smaller than symbol size. Regression lines are shown in red, green and blue colors for the Lower, Middle and Upper Ordovician, respectively. All Δ^{199}Hg and δ^{202}Hg data for the Wangjiawan section are from Gong et al.[70].

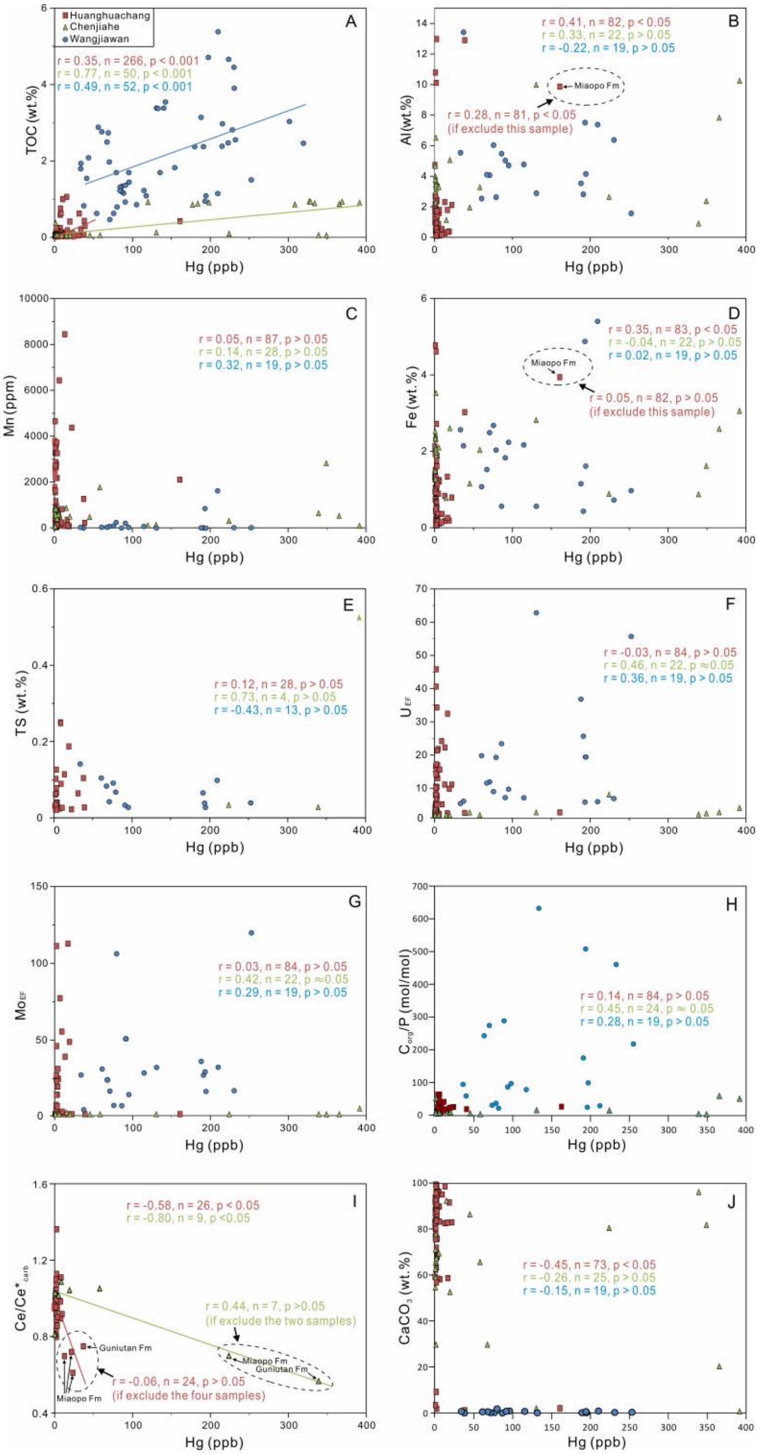

Supplementary Figure 11. Crossplots of (A) TOC vs. Hg, (B) Al vs. Hg, (C) Mn vs. Hg, and (D) Fe vs. Hg, (E) TS vs. Hg, (F) U_{EF} vs. Hg, (G) Mo_{EF} vs. Hg, (H) C_{org}/P vs. Hg, (I) Ce/Ce* vs. Hg, and (J) $CaCO_3$ vs. Hg for the study sections. *r* represents Pearson's correlation coefficient, *p* is the significance level, and *n* is the number of samples for each site. In panels B, D and I, the relatively high *r* values in the Miaopo and Guniutan formations are ascribed to a few samples with high Hg content, and exclusion of these samples from the statistical analysis leads to no or weak correlations.

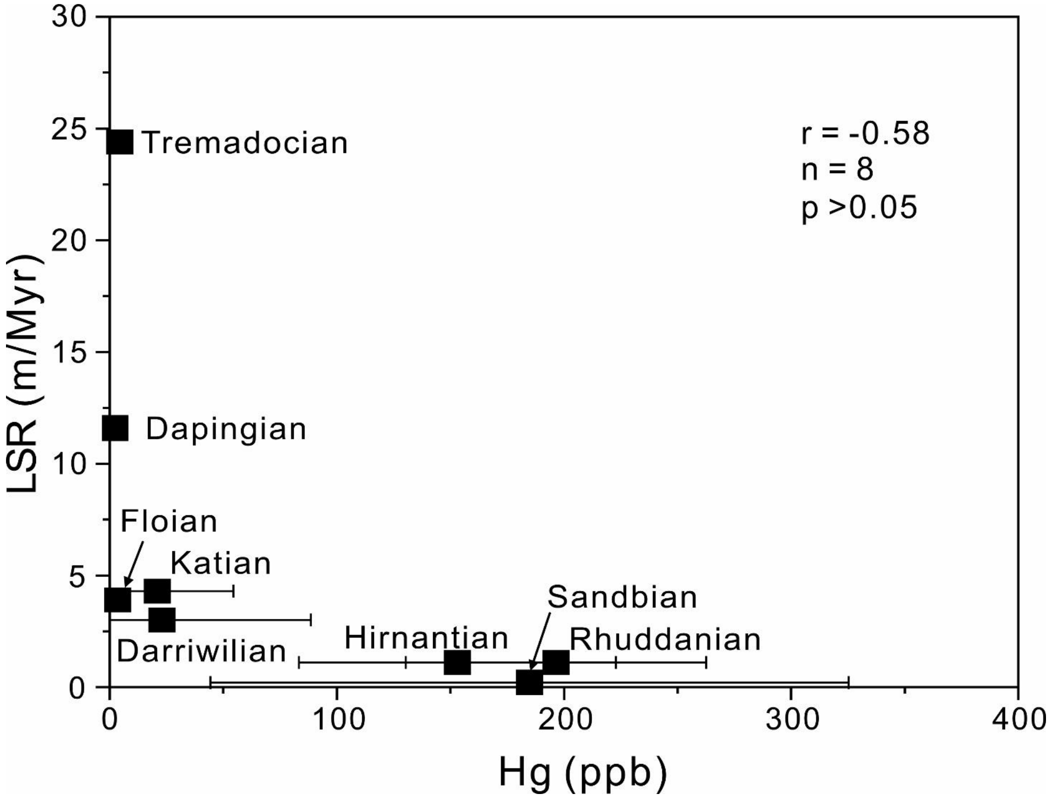

Supplementary Figure 12. Crossplots of linear sedimentation rates (LSR) vs. Hg for the study sections. *r* represents Pearson's correlation coefficient, *p* is the significance level, and *n* is the number of samples for each site. The LSR and Hg concentration data are average values for each formation based on three study sections.

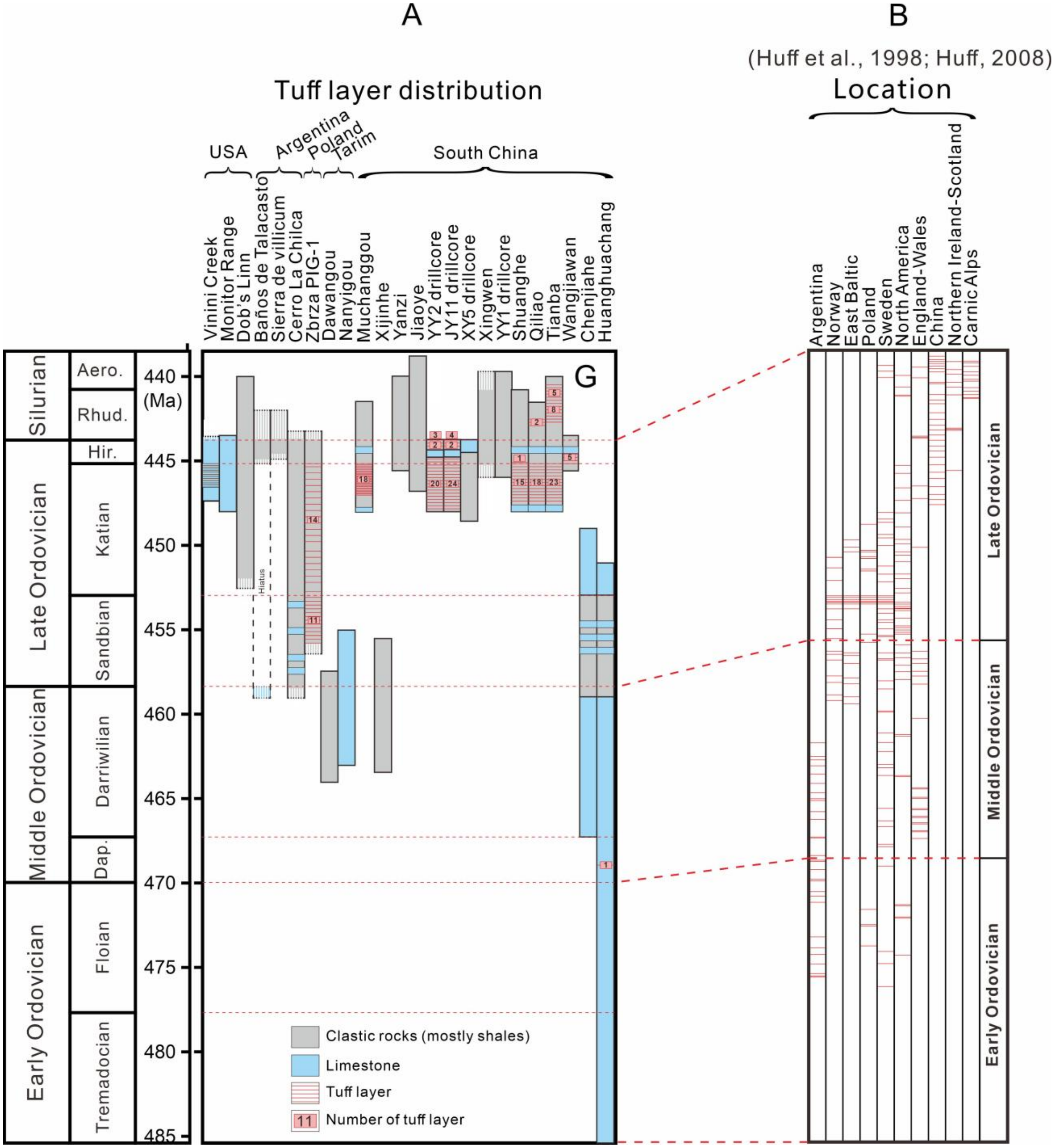

Supplementary Figure 13. Global comparison of temporal distribution of Ordovician volcanic tuff layers between our newly compiled dataset and previously published data. Published volcanic tuff layer data from Huff et al. [37,40]. Panel A is the same as Supplementary Fig. 8G.

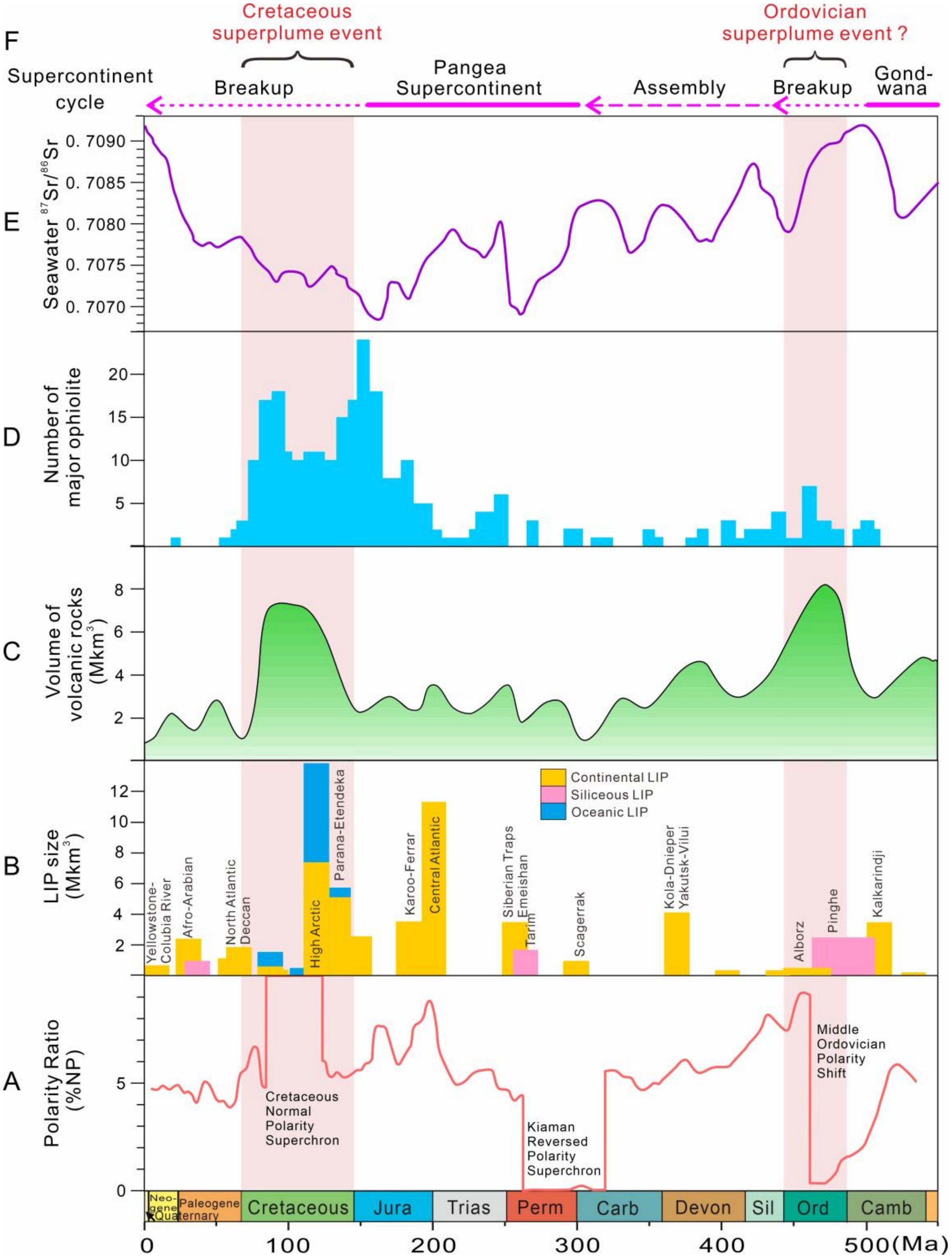

Supplementary Figure 14. Geological records for the Phanerozoic, showing secular volcanic activity through the Ordovician. (A) Magnetic polarity ratio (%NP = percent normal polarity)[71]; (B) Large igneous province (LIP) size[72-74]; (C) Volume of volcanic rocks[75]; (D) Ophiolite frequency[76]; (E) Seawater $^{87}Sr/^{86}Sr$[55]; (F) Supercontinent cycle[77].

Supplementary References

1 Wang, X. *et al.* The Global Stratotype Section and Point for the base of the Middle Ordovician Series and the third stage (Dapingian): Episodes, v. 32. *Biostratigraphy and Chronostratigraphy of the Great American Carbonate Bank* **135**, 987-1003 (2009).

2 Wang, X., Xiang, L. & Ni, S. Biostratigraphy of the Yangtze Gorge area (2): early Palaeozoic era. (1987).

3 Wang, Z., Zhen, Y., Ma, X. & Zhang, Y. Ordovician conodonts from the Kuniantan Topa Formation at Chenjihe and Zhenjin, Yichang, Hubei Province, China and their stratigraphic significance. *Acta Micropalaeontologica Sinica* **35**, 17 (2018).

4 Chen, X. *et al.* A global correlation of biozones across the Ordovician-Silurian boundary. *Acta Palaeontologica Sinica* **39**, 114-118 (2000).

5 Zhang, Y. *et al.* Ordovician integrative stratigraphy and timescale of China. *Science China Earth Sciences* **62**, 61-88 (2019).

6 Cramer, B. & Jarvis, I. in *Geologic Time Scale 2020* 309-343 (Elsevier, 2020).

7 Gorjan, P., Kaiho, K., Fike, D. A. & Xu, C. Carbon- and sulfur-isotope geochemistry of the Hirnantian (Late Ordovician) Wangjiawan (Riverside) section, South China: Global correlation and environmental event interpretation. *Palaeogeography Palaeoclimatology Palaeoecology* **337-338**, 14-22 (2012). https://doi.org:10.1016/j.palaeo.2012.03.021

8 Zhang, L. *et al.* Raman spectral, elemental, crystallinity, and oxygen-isotope variations in conodont apatite during diagenesis. *Geochimica et Cosmochimica Acta* **210**, 184-207 (2017).

9 Edwards, C. T., Jones, C. M., Quinton, P. C. & Fike, D. A. Oxygen isotope ($\delta^{18}O$) trends measured from Ordovician conodont apatite using secondary ion mass spectrometry (SIMS): Implications for paleo-thermometry studies. *GSA Bulletin* **134**, 261-274 (2022).

10 Albanesi, G. L., Barnes, C. R., Trotter, J. A., Williams, I. S. & Bergström, S. M. Comparative Lower-Middle Ordovician conodont oxygen isotope palaeothermometry of the Argentine Precordillera and Laurentian margins. *Palaeogeography Palaeoclimatology Palaeoecology* **549**, 109115 (2020).

11 Männik, P., Lehnert, O., Nolvak, J. & Joachimski, M. M. Climate changes in the pre-Hirnantian Late Ordovician based on $\delta^{18}O_{phos}$ studies from Estonia. *Palaeogeography Palaeoclimatology Palaeoecology* **569**, 110347 (2021).

12 Avila, T. D. *et al.* Role of seafloor production versus continental basalt weathering in Middle to Late Ordovician seawater $^{87}Sr/^{86}Sr$ and climate. *Earth and Planetary Science Letters* **593**, 117641 (2022).

13 Liu, K., Jiang, M., Zhang, L. & Chen, D. A new high-resolution palaeotemperature record during the Middle–Late Ordovician transition derived from conodont $\delta^{18}O$ palaeothermometry. *Journal of the Geological Society of London* **179**, jgs2021-2148 (2022).

14 Trotter, J. A., Williams, I. S., Barnes, C. R., Lécuyer, C. & Nicoll, R. S. Did cooling oceans trigger Ordovician biodiversification? Evidence from conodont thermometry. *Science* **321**, 550-554 (2008).

15 Trotter, J. A. & Eggins, S. M. Chemical systematics of conodont apatite determined by laser ablation ICPMS. *Chemical Geology* **233**, 196-216 (2006).

16 Wheeley, J. R., Jardine, P. E., Raine, R. J., Boomer, I. & Smith, M. P. Paleoecologic and paleoceanographic interpretation of $\delta^{18}O$ variability in Lower Ordovician conodont species. *Geology* **46**, 467-470 (2018). https://doi.org:10.1130/g40145.1

17 Löfgren, A. M. & Tolmacheva, T. J. Taxonomy and distribution of the Ordovician conodont *Drepanodus arcuatus* Pander, 1856, and related species. *Palaontologische Zeitschrift* (2003).

18 Stouge, S. S. Conodonts of the Middle Ordovician Table Head Formation, western Newfoundland. *Fossils and Strata* **16** (1984).

19 Pohler, S. Conodont biofacies of lower to lower middle ordovician megaconglomerates, Cow Head group, western Newfoundland. *Geological Survey of Canada Bulletin* **459**, pp. 71 (1994).

20 Jing, X., Zhou, H. & Wang, X. Biostratigraphy and biofacies of the Middle Darriwilian (Ordovician) conodonts from the Laoshidan section in the western margin of the North China Craton. *Marine Micropaleontology* **125**, 51-65 (2016). https://doi.org:10.1016/j.marmicro.2016.03.002
21 Jin, J., Zhan, R. & Wu, R. Equatorial cold-water tongue in the Late Ordovician. *Geology* **46**, 759-762 (2018). https://doi.org:10.1130/g45302.1
22 Trotter, J. A., Williams, I. S., Nicora, A., Mazza, M. & Rigo, M. Long-term cycles of Triassic climate change: a new $\delta^{18}O$ record from conodont apatite. *Earth and Planetary Science Letters* **415**, 165-174 (2015). https://doi.org:10.1016/j.epsl.2015.01.038
23 Bond, D. P. & Grasby, S. E. Late Ordovician mass extinction caused by volcanism, warming, and anoxia, not cooling and glaciation. *Geology* **48**, 777-781 (2020).
24 Finnegan, S. *et al.* The magnitude and duration of Late Ordovician–Early Silurian glaciation. *Science* **331**, 903-906 (2011).
25 Pucéat, E. *et al.* Revised phosphate–water fractionation equation reassessing paleotemperatures derived from biogenic apatite. *Earth and Planetary Science Letters* **298**, 135-142 (2010).
26 Scotese, C. R., Song, H., Mills, B. J. W. & van der Meer, D. G. Phanerozoic paleotemperatures: The earth's changing climate during the last 540 million years. *Earth-Science Reviews* **215** (2021). https://doi.org:10.1016/j.earscirev.2021.103503
27 Goldberg, S. L., Present, T. M., Finnegan, S. & Bergmann, K. D. A high-resolution record of early Paleozoic climate. *Proceedings of the National Academy of Sciences (U.S.A.)* **118**, e2013083118 (2021).
28 Liu, K., Jiang, M., Zhang, L. & Chen, D. A new high-resolution palaeotemperature record during the Middle–Late Ordovician transition derived from conodont $\delta^{18}O$ palaeothermometry. *Journal of the Geological Society* **179**, jgs2021-2148 (2022).
29 Charbonnier, G., Adatte, T., Föllmi, K. B. & Suan, G. Effect of Intense Weathering and Postdepositional Degradation of Organic Matter on Hg/TOC Proxy in Organic-rich Sediments and its Implicationsfor Deep-Time Investigations. *Geochemistry Geophysics Geosystems* **21** (2020). https://doi.org:10.1029/2019gc008707
30 Grasby, S. E., Them, T. R., II, Chen, Z., Yin, R. & Ardakani, O. H. Mercury as a proxy for volcanic emissions in the geologic record. *Earth-Science Reviews* **196**, 102880 (2019).
31 Chen, D., Ren, D., Deng, C., Tian, Z. & Yin, R. Mercury loss and isotope fractionation during high-pressure and high-temperature processing of sediments: Implication for the behaviors of mercury during metamorphism. *Geochimica et Cosmochimica Acta* **334**, 231-240 (2022).
32 Liu, Z., Tian, H., Yin, R., Chen, D. & Gai, H. Mercury loss and isotope fractionation during thermal maturation of organic-rich mudrocks. *Chemical Geology* (2022).
33 Zhang, L. *et al.* Diagenetic uptake of rare earth elements by conodont apatite. *Palaeogeography Palaeoclimatology Palaeoecology* **458**, 176-197 (2016).
34 Fendley, I. M. *et al.* Early Jurassic large igneous province carbon emissions constrained by sedimentary mercury. *Nature Geoscience* **17**, 241-248 (2024). https://doi.org:10.1038/s41561-024-01378-5
35 Racki, G., Rakociński, M., Marynowski, L. & Wignall, P. B. Mercury enrichments and the Frasnian-Famennian biotic crisis: A volcanic trigger proved? *Geology* **46**, 543-546 (2018). https://doi.org:10.1130/g40233.1
36 Liu, M. *et al.* Mercury isotope evidence for Middle Ordovician photic-zone euxinia: Implications for termination of the Great Ordovician biodiversification event. *Gondwana Research* **137**, 131-144 (2025). https://doi.org:10.1016/j.gr.2024.09.008
37 Huff, W. D. Ordovician K-bentonites: Issues in interpreting and correlating ancient tephras. *Quaternary International* **178**, 276-287 (2008).
38 Yang, S. *et al.* Globally synchronous meteorite rain during the Middle Ordovician. *Palaeogeography Palaeoclimatology Palaeoecology* **655** (2024). https://doi.org:10.1016/j.palaeo.2024.112550

39 Lu, Y. *et al.* Seawater sources of Hg enrichment in Ordovician-Silurian boundary strata, South China. *Palaeogeography Palaeoclimatology Palaeoecology* **601** (2022). https://doi.org:10.1016/j.palaeo.2022.111156
40 Huff, W. D., Bergström, S. M., Kolata, D. R., Cingolani, C. A. & Astini, R. A. Ordovician K-bentonites in the Argentine Precordillera: relations to Gondwana margin evolution. *Geological Society of London, Special Publication* **142**, 107-126 (1998). https://doi.org:10.1144/gsl.Sp.1998.142.01.06
41 German, C. R. & Elderfield, H. Application of the Ce anomaly as a paleoredox indicator: the ground rules. *Paleoceanography* **5**, 823-833 (1990).
42 Algeo, T. J. & Ingall, E. Sedimentary C_{org}: P ratios, paleocean ventilation, and Phanerozoic atmospheric pO_2. *Palaeogeography Palaeoclimatology Palaeoecology* **256**, 130-155 (2007).
43 Algeo, T. J. & Li, C. Redox classification and calibration of redox thresholds in sedimentary systems. *Geochimica et Cosmochimica Acta* **287**, 8-26 (2020).
44 Su, W. *et al.* K-bentonite, black-shale and flysch successions at the Ordovician–Silurian transition, South China: Possible sedimentary responses to the accretion of Cathaysia to the Yangtze Block and its implications for the evolution of Gondwana. *Gondwana Research* **15**, 111-130 (2009).
45 Su, W., He, L., Wang, Y., Gong, S. & Zou, H. K-bentonite beds and high-resolution integrated stratigraphy of the uppermost Ordovician Wufeng and the lowest Silurian Longmaxi formations in South China. *Science in China (Earth Sciences)* **46**, 1121-1133 (2003).
46 Su, W. *et al.* K–bentonite beds near the Ordovician–Silurian boundary on the Yangtze Platform, South China: preliminary study of the stratigraphic and tectonomagmatic significance. *Serie Correlación Geológica* **17**, 34 (2003).
47 Wang, L., Lin, S. & Xiao, W. Yangtze and Cathaysia blocks of South China: Their separate positions in Gondwana until early Paleozoic juxtaposition. *Geology* **51**, 723-727 (2023). https://doi.org:10.1130/g51362.1
48 Gernon, T. M. *et al.* Global chemical weathering dominated by continental arcs since the mid-Palaeozoic. *Nature Geoscience* **14**, 690-696 (2021). https://doi.org:10.1038/s41561-021-00806-0
49 Mason, E., Edmonds, M. & Turchyn, A. V. Remobilization of crustal carbon may dominate volcanic arc emissions. *Science* **357**, 290-294 (2017).
50 Zhang, M., Xu, S. & Sano, Y. Deep carbon recycling viewed from global plate tectonics. *National Science Review* **11** (2024). https://doi.org:10.1093/nsr/nwae089
51 Lee, C. T. A. *et al.* Continental arc-island arc fluctuations, growth of crustal carbonates, and long-term climate change. *Geosphere* **9**, 21-36 (2012). https://doi.org:10.1130/ges00822.1
52 McKenzie, N. R. *et al.* Continental arc volcanism as the principal driver of icehouse-greenhouse variability. *Science* **352**, 444-447 (2016).
53 Sternai, P. *et al.* Magmatic forcing of Cenozoic climate? *Journal of Geophysical Research: Solid Earth* **125**, e2018JB016460 (2020).
54 Saltzman, M. R. *et al.* Calibration of a conodont apatite-based Ordovician $^{87}Sr/^{86}Sr$ curve to biostratigraphy and geochronology: Implications for stratigraphic resolution. *GSA Bulletin* **126**, 1551-1568 (2014).
55 McArthur, J., Howarth, R., Shields, G. & Zhou, Y. in *Geologic Time Scale 2020* 211-238 (Elsevier, 2020).
56 Algeo, T. J., Marenco, P. J. & Saltzman, M. R. Co-evolution of oceans, climate, and the biosphere during the `Ordovician Revolution': A review. *Palaeogeography Palaeoclimatology Palaeoecology* **458**, 1-11 (2016).
57 Rubinstein, C. V., Gerrienne, P., de la Puente, G. S., Astini, R. A. & Steemans, P. Early Middle Ordovician evidence for land plants in Argentina (eastern Gondwana). *New Phytologist* **188**, 365-369 (2010).
58 Volkheimer, W. in *Proceedings of the IV International Palynology Conference Lucknow, India (Birbal*

Sahni Institute of Palaeobotany) 1976-1977.
59 Wellman, C. H. & Gray, J. The microfossil record of early land plants. *Philosophical Transactions of the Royal Society B Biological Sciences* **355**, 717-732 (2000).
60 Vavrdová, M. Coenobial acritarchs and other palynomorphs from the Arenig/Llanvirn boundary, Prague basin. *Vestnik Ustredniho Ustavu Geologickeho* **65**, 237-242 (1990).
61 Vavrdová, M. Some plant microfossils of possible terrestrial origin from the Ordovician of Central Bohemia. *Vestnik Ustredniho Ustavu Geologickeho* **59**, 165-170 (1984).
62 Strother, P. K., Al-Hajri, S. & Traverse, A. New evidence for land plants from the lower Middle Ordovician of Saudi Arabia. *Geology* **24**, 55 (1996).
63 Philippe *et al.* Origin and radiation of the earliest vascular land plants. *Science* **324**, 353 (2009).
64 Badawy, A. S., Mehlqvist, K., Vajda, V., Ahlberg, P. & Calner, M. Late Ordovician (Katian) spores in Sweden: oldest land plant remains from Baltica. *Geologiska Fã¶reningen I Stockholm Fã¶rhandlingar* **136**, 16-21 (2014).
65 Redecker & D. Glomalean Fungi from the Ordovician. *Science* **289**, 1920-1921 (2000).
66 Wellman, C. H., Osterloff, P. L. & Mohiuddin, U. Fragments of the earliest land plants. *Nature* **425**, 282-285 (2003).
67 Steemans, P. *et al.* Origin and radiation of the earliest vascular land plants. *Science* **324**, 353-353 (2009).
68 Lenton, T. M., Crouch, M., Johnson, M., Pires, N. & Dolan, L. First plants cooled the Ordovician. *Nature Geoscience* **5**, 86-89 (2012).
69 Chen, X. *et al.* The Global Boundary Stratotype Section and Point (GSSP) for the base of the Hirnantian Stage (the uppermost of the Ordovician System). *Episodes* **29**, 183-196 (2006).
70 Gong, Q. *et al.* Mercury spikes suggest volcanic driver of the Ordovician-Silurian mass extinction. *Scientific Reports* **7**, 5304 (2017).
71 Algeo, T. J. Geomagnetic polarity bias patterns through the Phanerozoic. *Journal of Geophysical Research: Solid Earth* **101**, 2785-2814 (1996).
72 Nechaev, V. P., Sutherland, F. L. & Nechaeva, E. V. Phanerozoic evolution of continental large igneous provinces: Implications for galactic seasonality. *Minerals* **12**, 1150 (2022).
73 Dan, W. *et al.* Cambrian–Ordovician magmatic flare-up in NE Gondwana: A silicic large igneous province? *GSA Bulletin* **135**, 1618-1632 (2023).
74 Derakhshi, M., Ernst, R. E. & Kamo, S. L. Ordovician-Silurian volcanism in northern Iran: Implications for a new Large Igneous Province (LIP) and a robust candidate for the Late Ordovician mass extinction. *Gondwana Research* **107**, 256-280 (2022).
75 Ronov, A. Stratisfera ili Osadochnaya Obolochka Zemli: Kolichestvennoye Issledovanie (Stratisphere, or the Sedimentary Sphere of the Earth: Quantitative Research). *Moscow (in Russian)* (1993).
76 Dilek, Y. & Newcomb, S. Ophiolite concept and its evolution. *Geological Society of America, Special Publication* **373**, 1-16 (2003).
77 Nance, R. D., Murphy, J. B. & Santosh, M. The supercontinent cycle: a retrospective essay. *Gondwana Research* **25**, 4-29 (2014).
